# Assessing the status of iodine deficiency disorder (IDD) and associated factors in Wolaita and Dawro Zones School Adolescents, southern Ethiopia

**DOI:** 10.1186/s13104-017-2480-5

**Published:** 2017-04-18

**Authors:** Shimelash Bitew Workie, Yemane Gebremariam Abebe, Amha Admasie Gelaye, Tefera Chane Mekonen

**Affiliations:** 1 College of Health Science and Medicine, Wolaita Sodo University, Wolayta Sodo, Ethiopia; 2 College of Health Science and Medicine Mekele University, Mekelle, Ethiopia; 3 College of Health Science and Medicine Wollo University, Dessie, Ethiopia

**Keywords:** Iodine deficiency, Adolescent students, Wolaita and Dawuro zone, Ethiopia

## Abstract

**Background:**

Iodine deficiency is the major preventable cause of irreversible mental retardation in the world. Ethiopia is a country with high prevalence of iodine deficiency disorders which continue to affect a large number of the country’s population. The aim of the study was to assess the prevalence of iodine deficiency disorder in Wolaita and Dawuro zones.

**Methods:**

A descriptive, cross-sectional study was conducted in high school and preparatory students in Wolaita and Dawuro zones between April and May 2012. Data were collected from 718 school adolescents using pre-tested questionnaire through systematic random sampling technique. Data were entered and cleaned using Epi-info version 3.5.3 and then transported to SPSS version 20 for analysis. Bivariate and Multivariable logistic regression were done and the cut off value set was P < 0.05 as this is considered as statistically significant.

**Result:**

The overall prevalence (total goiter rate) of goiter in study area was 351 (48.9%). Students with Grade-1 goiter were 265 (36.9%) while with Grade-2 goiter was 86 (11.9%). Females were by a long way vulnerable for goiter and accounts 60.9% with Pearson correlation coefficient 0.300, P value 0.0001. Generally, the occurrence of goiter in the study area was found to have statistical significant association with sex of respondents (being female) [AOR = 3.526; 95% CI (2.55–4.87)], climatic condition of resident (temperate climate) [AOR = 0.617; 95% CI (0.404–0.943)], frequency of iodized salt use [AOR = 0.484; 95% CI (0.317–0.739)] and consumption of cassava [AOR = 4.184; 95% CI (2.6–6.707)].

**Conclusion and recommendation:**

In general, the study revealed that iodine deficiency disorder was a serious public health concern. Nearly half of adolescent students in Wolaita and Dawuro zones were affected by goiter. Therefore, emphasis on a sustainable iodine intervention program targeted at population level, particularly at females is mandatory. Nutrition education along with adequate Universal Salt Iodization program is urgently required.

## Background

Iodine deficiency disorder, one of the most prevalent micronutrient deficiencies globally, is the main cause of potentially preventable mental retardation in childhood, as well as a spectrum of morbidities referred to as iodine deficiency disorders (IDD). The World Health Organization estimates that approximately 37% of school-age children, 285 million, and 1.88 billion people worldwide remain at risk of insufficient iodine intake and approximately a third of the world’s population lives in areas with some iodine deficiency [1]. Iodine deficiency is not only a common problem of developing countries but also developed countries including Australia, New Zealand and the United Kingdom are confronted with re-emergence of mild iodine deficiency and taken as public health problem in more than 50 countries [2, 3].

The estimated annual potential cost attributable to IDD in the developing world prior to widespread salt iodization was $35.7 billion per year versus $0.5 billion per year after salt iodization, giving a benefit–cost ratio of 70:1 [4].

Iodine deficiency poses a threat throughout the life-cycle and has been associated with mental impairment and goitre in older children and adults and complications with pregnancy, including stillbirth and congenital anomalies [5]. Inadequate iodine intake during pregnancy may lead to irreversible foetal brain damage. Ethiopia is a country with a high prevalence of iodine deficiency disorders which continue to affect a large number of the country’s population. Goiter prevalence rates vary significantly from region to region in Ethiopia and in certain areas the prevalence rate may be as high as 71% [6]. Total goiter prevalence (weighted) in Ethiopia was 35.8% in which 24.3 and 11.5% were palpable and visible goiter respectively. Goiter prevalence in four regional states namely Southern Nation Nationalities and People Region (SNNPR), Oromia, Benshangul-Gumuz and Tigray was greater than 30%, with the maximum of about 60% in SNNPR, an indication of severe iodine deficiency [7].

Due to the severity of IDD in the nation, Ethiopia mandated that all salt for human consumption should be iodized since February 2011. A national survey conducted in 2014 by Ethiopia’s Public Health Institute (EPHI) found that an impressive 95% of households were using iodized salt. However, only 43% of the salt contained more than 15 ppm of iodine [8].

In the past 5 or more years, different small-scale studies or pocket studies and two nationwide studies conclude that Ethiopia is suffering from severe iodine deficiency as using total goitre rate as indicator and in all studies the prevalence was more than 30% [9–11]. This indicates that the presence of severe iodine deficiency accomplished with cretinism, poor scholastic performance and reduced economic productivity in general.

The problem of iodine insufficiency had got major public concern in the country and legislation as well as specific intervention strategies has been developed and implemented throughout the nation. But currently there are no scientific evidences that whether the implemented interventions are effectively reduce total goitre rate. In addition, from nationwide survey report the highest goitre rate (56%) was observed in southern Ethiopia, the area where consumption of cassava and other iodine intake inhibitor diets are very common [6]. The goiter prevalence in Ethiopia especially in SNNPR is a big problem. The magnitude of goiter among adolescent students in Ethiopia, have not been well assessed. It was necessary and timely to study this important issue in the adolescent age group. This study was therefore aimed at investigating the magnitude of goiter and associated risk factors in Wolaita and Dawuro zones, Southern Ethiopia that would serve as a baseline data.

## Methods

### Study area and period

The study was conducted from April to May, 2012, in Wolaita and Dawuro Zones. Wolaita zone is one of the thirteen zones of the SNNPR region covering an area of 4471.3 km^2^. For administrative purpose, it is divided into twelve Woredas and three administrative cities. Topographically the zone lies on an elevation ranging from 1200 to 2950 m above sea level. The zone has three agro-ecological zones. Dega (cold climate) (3%) Woina dega (temperate climate) (57%) and Kolla (hot climate) (40%). The annual average temperature of the zone is 15.1 °C and the mean annual rainfall ranges from 1200 to 1300 mm. Sodo town is the administrative center of the zone. It is located at a distance of 380 km. South of Addis Ababa and 157 km away from Hawassa town.

Dawuro zone is also another zone in SNNPR region and administratively divided into five Districts and one administrative city. Topographically the zone lies on an elevation ranging from 500 to 2800 m above sea level. The zone has three agro-ecological zones. It is located at a distance of 497 km south of Addis Ababa and 274 km away from Hawassa town.

### Study design

An institutional based cross-sectional study design was employed.

### Source population

All high school and preparatory adolescents were involved in two zones.

### Study population

Participants (high school and preparatory students) or study subjects were selected from the selected city administration of the two zones using a purposive sampling technique.

### Sample size determination

The required sample size of the study was determined by single population proportion formula $n = \frac{{z^{2} \left( {\alpha |2} \right) \times P\left( {1 - P} \right)}}{{d^{2} }}$ as 59.1% prevalence of goiter in school of Shebe Senbo District [11] is used to estimate the sample size, with a margin of error 4%, confidence interval of 95%. Final sample was multiplied by design effect of 2 and the total sample size was 740.

### Sampling procedure

The sampling procedure was multi stage sampling method. First four city administrations from the two zones were purposely selected. All high schools and preparatory schools were selected which is found in all city administrations. Then sample size was allocated to each school based on the number of students found in each school and grade. Students list was found from the school registrar. Finally, systematic random sampling technique was employed to select each student.

### Data collection instrument and procedure

Data was collected using structured self-administered questionnaires. Clinical assessment of goiter was made by five health officers. The stage of the thyroid gland enlargement examination was graded on standard survey forms. Physical examination of the thyroid gland was done to assess goiter rate using the WHO/UNICEF/ICCIDD classification scheme [5, 12]. The gland classified as grade 0: Normal (No palpable or no visible), Grade 1: goiter palpable, in normal position and Grade 2: goiter visible in normal position; and total goiter prevalence (TGP) was measured by the sum of Grade 1 and Grade 2.

The principal investigators trained five health officers for clinical diagnosis of goiter and ten nurses for data collection activity from Wolaita Sodo University for one day on instruction and how to supervise the whole activity.

The English version of the questionnaire was translated to local (Amharic) language.

The supervisors clarified any doubts and collected the questionnaire after filled by the respondents.

General safety procedures during diagnosis were applied and special or separate class room for diagnosing students used in school, this helped students felt free to show their neck.

### Data quality control

To assure the quality of the data in the study, data collectors and supervisors were trained and a regular supervision and follow-up was made by supervisors and the principal investigator. In addition, regular check-up for completeness and consistency of the data were made on daily basis. English version questionnaire was translated to Amharic and back translated to English by translators who are blind to the original questionnaire. To assure the quality of the data high emphasis was given in designing data collection instrument for its simplicity and pre-test followed by modification was made. Prior to the data collection, pre-test was conducted on 5% of the total sample size of the respondent’s from Humbo Woreda of the two zones.

### Data analysis procedures

After thorough check up for completeness, free from any error on daily, the data were coded, cleaned and entered into Epi-info version 3.5.3 and exported to SPSS version 20 for analysis. Then descriptive frequencies were used for checking of outliers and to clean the data. The frequency distribution of dependent and independent variables was worked out. Correlation, Bivariate and Multivariable logistic regression were done. For all statistical significance test, the cut off value set was P < 0.05 as this is considered statistically reliable for the analysis of this study.

## Results

### Socio-demographic characteristics

Of the total 740 sample size, a complete response was obtained from 718, which makes 97.0% response rate. From the total study participants, 390 (54.3%) were males and 328 (45.7%) were females resulting in an overall male to female ratio of 1.18:1. Majority of the students, 607 (84.5%) were between the ages of 15 and 19 years and 654 (91.1%) were never married.

Out of the total 718 students, 64.5% were lived in plain land topography type while the rest were in mountainous type. Four hundred twenty-one (58.6%) of the study participants came from urban places (Table 1).

**Table 1:** Socio demographic characteristics of high school and preparatory students in Wolaita and Dawuro zones, Southern Ethiopia, April, 2012

Variable	Number (n = 718)	Percent
Sex of the student		
Male	390	54.3
Female	328	45.7
Age of the students (years)		
10–14	52	7.3
15–19	607	84.5
20+	59	8.2
Marital status		
Single	654	91.1
Married	47	6.5
Divorce and widowed	17	2.4
Religion		
Orthodox	230	32
Protestant	452	63.0
Other	36	5
Ethnics of the student		
Wolaita	517	72.0
Dawuro	159	22.1
Other (Amhara, Oromo …)	42	5.9
Permanent address of the student		
Urban	421	58.6
Rural	297	41.4
Climate condition		
Dega	273	38.0
Woina dega	283	39.4
Kola	162	22.6
Topography of the area		
Plain land	463	64.5
Mountainous	255	35.5
Family size (n = 574)		
<5 family	378	65.9
≥5 family	196	34.1

### Dietary habit of respondents

Salt utilization, half of the study participants were used non-iodized type (commonly known as rock salt) 361 (50.3%) and one quarter of the respondents were consumed iodized salt commonly known as table salt) 173 (24.1%). Among iodized salt users in their home, 160 (43.8%) of them used it always, while the rest has been used it sometimes and not at all.

There were different types of food items commonly eaten in the area. Among these fruits were predominantly consumed 409 (58.8%) and followed by others such as cereal, legume and tubers, but fish were rarely consumed in the area 69 (9.8%) only.

Among the students 584 (81.3%) of them ate Cassava and 326 (56%) of the students ate once in a week while 111 (19.1%) ate twice in a week. Similarly, 684 (95.26%) and 670 (93.3%) of them ate Cabbage and spinach (Habesha Gommen) respectively (Table 2).

**Table 2:** Iodized salt utilization and dietary intake characteristics of high school and preparatory students in Wolaita and Dawuro zones, Southern Ethiopian, April, 2012

Variables	Frequency (718)	Percent
Type of salt frequently used		
Rock salt (non iodized)	361	50.7
Iodized (table salt)	173	24.1
Both	181	25.2
How often you use iodized table salt in your home?		
Not use	337	46.9
Always	153	21.3
Sometimes	228	31.8
Source of iodized salt (n = 365)		
Shop/market	314	86
Donation	51	14
Is iodized salt available at the source? (n = 363)		
Yes	194	53.4
No	169	46.6
Type of food frequently eaten		
Cereal (n = 703)	345	49.1
Legume (n = 703)	348	49.5
Tuber (n = 703)	328	46.7
Fruit (n = 701)	409	58.3
Fish (n = 703)	69	9.8
Others (n = 703)	224	31.9
Did you eat cassava?		
Yes	584	81.3
No	134	18.7
When did you consume cassava? (584)		
Only during summer	229	39.3
Every time during food shortage	144	24.8
When we want to eat it	176	30.4
Other	53	9.2
How frequently consume cassava per week?		
Not eat cassava	134	18.7
Only 1 day	290	40.4
2 days	108	15.4
3 days	76	10.6
4 or more days	110	15.3
In what form did you consume cassava? (n = 584)		
Only boiled	233	39.8
In injera/porridge/bread/possessed	156	26.8
Both	195	33.4
Did you consume cabbage?		
Yes	684	95.26
No	34	4.74
When did you consume cabbage? (684)		
Only during summer	165	24.4
Every time during food shortage	107	15.9
When we want to eat it	342	50.5
Other	85	12.6
How frequently did you consume cabbage?		
Not eat	34	4.74
Only 1 day	180	26.7
2 days	202	30
3 days	93	13.8
4 or more days	199	29.5
Did you consume Habesha Gommen?		
Yes	670	93.3
No	48	6.7
When did you consume Habesha Gommen? (670)		
Only during summer	173	25.8
Every time during food shortage	307	45.8
When we want to eat it	149	22.2
Other	60	8.95
How frequently did you consume Habesha Gommen? (n = 668)		
Only 1 day	155	23.2
2 days	150	22.5
3 days	125	18.7
4 and more days	238	35.6

### Knowledge about goiter among students

Six hundred twenty-two, (90.5%) of the study participants had ever heard about goiter. Among these 29.3, 22, 20.1, 16.4% of the participant reported that the causes of goiter were inadequate dietary intake, drinking unprotected water, eating goiter causing foods and family predisposition respectively.

Concerning the health risks of goiter other than goiter, 500 (79.7%) of the respondent believed that goiter can cause other health risk, while the rest 86 (13.7%) and 41 (6.1%) they didn’t know about other risks and believed no other causes respectively. Among the potential risks of goiter, 252 (49.3%) of the respondent believed that goiter is a risk to physical deficit and followed by reduce school performance 123 (24.1%).

Among the study participants, two hundred twelve (31.8%) of them had a goiter victim family member. Among these family only 131 (65.2%) did get treatment for the goiter. Regarding to preference for treatment of goiter, 117 (68%) of them were got treatment from health institution and followed by traditional healer 24 (14%) (Table 3).

**Table 3:** Knowledge of students about Goiter/IDDs of high school and preparatory students in Wolaita and Dawuro zones, Southern Ethiopia, April, 2012

Variables	Frequency	Percent
What are the causes of goiter?		
In adequate dietary intake (n = 627)	184	29.3
Eating goiter causing foods (n = 627)	126	20.1
Drinking unprotected water (n = 627)	138	22
Family predisposition (n = 627)	103	16.4
Supernatural being or evil spirit (n = 626)	33	5.3
Spontaneous event (n = 627)	33	5.3
Other (n = 627)	36	5.7
I don’t know the cause (n = 627)	93	14.8
Do you know the health risk of goiter? (n = 627)		
Yes	500	79.7
No	41	6.5
I don’t know	86	13.7
What are these risks of goiter?		
Reduce school performance (n = 510)	123	24.1
Physical deficit (n = 511)	252	49.3
Abortion or miscarriage (n = 511)	72	14.1
Social discrimination (n = 511)	76	14.9
Other (n = 511)	45	8.8
Do you think goiter is preventable? (n = 629)		
Yes	567	90.1
No	32	5.1
I don’t know	30	4.8
How do you think one can prevent from goiter?		
Using iodized table salt (n = 574)	394	68.6
Taking proper nutrition (n = 574)	127	22.1
Drinking safe water (n = 574)	117	20.4
Keeping sanitation (n = 574)	52	9.1
Avoiding sin or evil act (n = 574)	53	9.2
Other (n = 574)	14	2.4
Is their any one had goiter in your family? (n = 667)		
Yes	213	31.9
No	454	68.2
What is the relationship with you?		
Mother (n = 213)	42	19.7
Father (n = 213)	22	10.3
Garand parents (n = 213)	23	10.8
Brother or sister (n = 213)	59	27.8
Uncle (n = 213)	18	8.5
Aunt (n = 213)	27	12.7
Myself (n = 213)	20	9.4
Other (n = 213)	20	9.4
Did they get treatment? (n = 201)		
Yes	131	65.2
No	70	34.8
Where did they get treatment?		
Traditional healer (n = 172)	24	14
Health institution (n = 172)	117	68
Religious healer (n = 172)	22	12.8
Other (n = 172)	8	4.7

### Prevalence of goiter

Overall prevalence of goiter in study area was 48.9%. Status of goiter in the study area varied with its stage, goiter stage of Grade-1, 265 (36.9%) was higher than Grade-2 which was 86 (11.9%). Out of these female accounts 214 (60.9%) with Pearson correlation coefficient 0.30 P value 0.00001.

### Factors associated with goitre (thyroid gland enlargement)

In bivariate analysis of socio-demographic determinants being female is the risk for goiter compared with male with COR = 3.46 (95% CI 2.54–4.72) but age has no association with occurrence of goiter. Meanwhile, respondents who lived in the temperate (Woina Dega) climatic areas are a protective factor against the occurrence of goiter [COR = 0.62, (95% CI 0.42–0.91)]. The remaining socio-demographic variables had no association with occurrence of goiter.

From dietary pattern in bivariate analysis type of salt had no statistically association with goiter rather the frequency of iodized salt usage had an association with the occurrence of goitre. Iodized salt always user had preventive effect on the occurrence of goiter i.e. (COR 0.65, 95% CI 0.44–0.96). Those who ate cassava also had an effect on the occurrence of goitre with COR = 3.49 (95% CI 2.29–5.32) but frequency of eating cassava had no association with the occurrence of goiter. Prevalence of goiter has an association with eating of cabbage as compared to their counterparts. Similar to that of cassava frequency of cabbage eating had no association with occurrence of goiter. During multivariable logistic regression analysis, only sex of the respondent, climatic condition of the living area, frequency of iodized salt usage and eating of cassava had an association with the occurrence of goiter (Table 4).

**Table 4:** Association different factors on goiter status among high school and preparatory students in Wolaita and Dawuro zones, Southern Ethiopia, April 2012

Variables	Goiter status		Crude OR (95% CI)	Adjusted OD (95% CI)
	Yes	No		
Sex of the student				
Male	137	253	1.00	1.00
Female	214	114	3.46 (2.54, 4.72)*	3.526 (2.55–4.87)*
Age of students (years)				
10–14	20	32	1.00	
15–19	301	306	1.57 (0.88–2.8)	
20+	30	29	1.66 (0.78–3.528)	
Permanent address of the student				
Urban	197	224	0.81 (0.60, 1.10)	
Rural	154	143	1.00	1.00
Climate condition				
Dega	137	135	0.82 (0.55, 1.21)	0.775 (0.506–1.188)
Woina dega	122	159	0.62 (0.42, 0.91)*	0.617 (0.404–0.943)*
Kola	89	72	1.00	1.00
Topography of the area				
Plain land	227	234	1.06 (0.78, 1.45)	
Mountainous	122	132	1.00	
Type of salt used				
Non iodized (rock salt)	186	175	1.00	
Iodized (table salt)	73	100	0.696 (0.48–1.00)	
Both	91	90	0.93 (0.65–1.28)	
How often you use iodized table salt in your home?				
Not use	172	165	1.00	1.00
Always	62	91	0.65 (0.44–0.96)*	0.484 (0.317–0.739)*
Sometimes	117	111	1.01 (0.72–1.42)	0.876 (0.607–1.625)
Do you consume cassava?				
Yes	317	267	3.49 (2.29–5.32)*	4.184 (2.6–6.707)*
No	34	100	1.00	1.00
How frequently consume cassava per week?				
Not eat cassava	34	100	0.316 (0.184–0.54)*	
Only 1 day	154	136	1.05 (0.679–1.634)	
2 days	61	47	1.207 (0.708–2.057)	
3 days	45	31	1.35 (0.748–2.437)	
4 or more days	57	53	1.00	
Do you consume cabbage?				
Yes	331	353	1.000	
No	20	14	0.66 (0.328–1.32)	
How frequently did you consume cabbage?				
No eat	20	14	0.66 ( 0.312–1.38)	
Only 1 day	87	93	0.574 (0.275–1.199)	
2 days	91	111	0.685 (0.309–1.52)	
3 days	46	47	0.734 (0.352–1.53)	
4 or more days	107	102	1.00	
Family history of goiter				
Yes	104	109	1.003 (0.728–1.382)	
No	247	258	1.00	

* Significant P value <0.05

## Discussion

Overall prevalence of goiter among school adolescents was 48.9% in this area. The status of goiter in the study area varied with its stage, Grade-one goiter (36.9%) was higher than Grade-two which was (11.9%). Females were significantly affected (60.9%) as compared to males. The rate of goiter among adolescent students of Wolaita and Dawuro zones was found to be higher than report on global burden of Iodine deficiency accounted 16% of world population and 27% of African population [13] and finding from Western part of Germany (23.9%) [14]. Likewise, it is also higher than finding from Tanzania which is 25% of TGP in 6–18 years of school children [15]. However, it was less than findings from northwest (54%) [10] and southwest (59%) [11] Ethiopia; and in Enda-Mehoni district in Tigray, Ethiopia, 71.4% [16] and school children in Islamabad which is 71.6% [17]. The presence of high prevalence of goiter in this area may be due to low or inhibited thyroidal uptake of iodine because of frequent and high consumption of cassava and low coverage of iodized salt and poor utilization of iodized salt at household.

The study also revealed that sex of the students was significantly associated with goiter prevalence. This finding is supported by studies in Sub-Saharan Africa and northern Ethiopia which states that females were drastically affected [16, 18], and on the other hand, it is contradicted with findings from western part of Germany and Islamabad reported that males were more likely affected than females [14, 17]. In our finding the reason may be female adolescents have greater physiological demand of nutrients particularly iron and iodine due to the burden of menstruation and development of secondary sexual characteristics in addition with stigmatization or depravation due to sex preferences.

Regarding with climatic condition of the residents, participants from Woina Dega (temperate or medium climatic condition) were less likely to develop goiter as contrast to who lived in Dega (cold temperature) and kola (hot temperature). The result is consistent with findings from Veneto Region, Italy and in different geographical landscape of Ethiopia [12, 19]. Probably individual reside in hot temperature will suffer from inadequate intake of iodine due to its nature of volatility apart from the presence of other iodine inhibitors.

Despite of the availability or the presences of iodized salt, respondents who were always or frequently used iodized salt were protected from the occurrences of goiter as compared to those who used sometimes or never users. This is similar with report from Germany and Italy [14, 19]. This may be in the study setting daily intake of iodized salt may overcome the competitive inhibition of thyroidal uptake of iodine.

Most common staple diet of the participants was cassava that accounts about 82% and in this study, Cassava consumption was identified as independent predictors of goiter and the result was similar with study conducted in sub-Saharan Africa [18]. Cassava contains cyanide compound that compete for uptake by the gland. Therefore, high concentrations of cyanide considerably reduce the absorption of iodine and the gland progressively enlarged (hyperplasia of the gland).

Eventually, the study failed to measure the recent iodine level in the participants which is the drawback of the use of total goiter prevalence that is not sensitive indicators of IDD. Moreover, the study had limitation to generalized the overabundance of goiter is purely the consequence of iodine deficiency. The subjects under study were not the preferable target population to study iodine deficiency and there could have been a room for recall bias which subsequently could have resulted in underestimation of the true prevalence of goiter among the students.

## Conclusion

In general, goiter was a serious public health concern in both zones. Significant proportion of students in Wolaita and Dawuro zones, were affected by goiter and female students were getting serious impact than male students. Iodine salt utilization is not enough to prevent goiter unless it was used always. Generally, the occurrence of goiter was affected by sex, climatic condition, frequency of iodine salt utilization, and cassava consumption.

## Recommendation

Emphasis should be given on a sustainable iodine intervention program targeted at population particularly female. Nutrition education along with Universal Salt Iodization program where iodine deficiency is severe is urgently required. Intensive iodized salt donation to the community is the option until awareness of the community is optimum. It is very imperative that health education should be given to increase community awareness on the importance of iodized salt to the general public.

## Abbreviations

AOR: adjusted odds ratio
COR: crude odds ratio
IDD: iodine deficiency disorder
SNNPR: Southern Nations and Nationality Peoples’ Region
TGP: total goiter prevalence
